# Pregnancy Outcome After First‐Trimester Exposure to Statins: Study Results Based on the Embryotox Cohort

**DOI:** 10.1002/pds.70481

**Published:** 2026-09-28

**Authors:** Evelin Beck, Andrea T. Adelsberger, Katarina Dathe

**Affiliations:** ^1^ Charité—University Medical Center Berlin, Corporate Member of Freie Universität Berlin and Humboldt‐Universität zu Berlin, Institute of Clinical Pharmacology and Toxicology, Embryotox Centre of Clinical Teratology and Drug Safety in Pregnancy Berlin Germany

**Keywords:** abortions, spontaneous (MeSH), congenital abnormalities (MeSH), hydroxymethylglutaryl‐CoA reductase inhibitors (MeSH), pharmacovigilance (MeSH), pregnancy outcome (MeSH)

## Abstract

**Purpose:**

This observational cohort study aimed to assess the risk of adverse pregnancy and neonatal outcomes in pregnancies exposed to statins during the first trimester, given that the use of statins during pregnancy remains controversial. The primary study endpoints were the rates of spontaneous abortion and major birth defects.

**Methods:**

In total, 188 pregnancies exposed to statins in the first trimester were compared with a cohort of 564 pregnancies without the study medication, which were recorded at the Embryotox Centre between January 2000 and June 2021. The effect of statin exposure on pregnancy outcome was estimated using Cox proportional hazards regression models. Log‐binomial regression was used to evaluate the risk of major birth defects.

**Results:**

Major birth defects were observed in 10 of 135 (7.4%) infants in the statin cohort and in 12 of 501 (2.4%) infants in the comparison cohort (RR 3.09, 95% CI 1.37–7.00). After adjustment for relevant baseline covariates and additional maternal disease factors, the difference substantially attenuated (RRadj 2.16; 95% CI 0.88–5.31). The cumulative incidence of spontaneous abortion was 26.8% in the statin‐exposed versus 15.0% in the comparison cohort (HR 1.86, 95% CI 1.13–3.04; HRadj 1.66, 95% CI 0.99–2.78).

**Conclusions:**

Current evidence suggests that statins are not major teratogens. The observed associations between statin exposure and birth defects, as well as unfavourable pregnancy outcomes, are presumably influenced by confounding factors such as underlying maternal disease. Our study results lend support to this assumption. However, they should be interpreted cautiously due to the limited cohort size.

## Key Points

- Pregnant women who are treated with statins are likely to have an underlying disease risk profile that is higher than average. It is necessary to adjust for relevant baseline factors and maternal health status.
- Adverse pregnancy outcomes may be more strongly influenced by the underlying maternal medical condition than by statin treatment per se.
- A key strength of the analysed dataset is that it is based on comprehensive information on statin exposure during the first trimester and includes an assessment of birth defects in both live and non‐live births.
- Statin therapy during pregnancy, especially in the first trimester, should only be considered after carefully weighing the risks and benefits.

## 1 Purpose

Cholesterol plays a vital role in embryonic development, leading to long‐standing concerns about the use of HMG‐CoA reductase inhibitors, commonly known as statins, during pregnancy. Overall, the available data on statin treatment for pregnant women are inconsistent. However, in recent studies and meta‐analyses no significant association was observed between statins and birth defect rates [1, 2, 3]. The prevailing opinion is that statin treatment should be discontinued in most pregnant patients. In clinical practice, statins should only be considered in high‐risk situations where the therapeutic need clearly outweighs the potential risks. While the Food and Drug Administration (FDA) has supported such an approach in its regulatory guidelines since 2021, the EMA is stricter and still issues a general contraindication for use during pregnancy [4, 5].

This study analysed data that were systematically collected and archived in the German Embryotox database. The primary objectives of this study were to evaluate the risk of major birth defects and spontaneous abortion (SAB) following first‐trimester statin exposure.

## 2 Methods

### 2.1 Study Design and Definitions

This observational cohort study draws on archived pregnancy data from the consultation and counselling service of the German Embryotox Centre. A structured approach was followed to obtain relevant data during the first consultation request, including information on drug exposure, indications, maternal characteristics and obstetric history. Approximately 8 weeks after the estimated date of birth a follow‐up questionnaire was sent including questions on maternal drug use, pregnancy complications, neonatal outcome and congenital anomalies. Additional inquiries followed in cases of implausible or insufficient information. All data were recorded in the Embryotox database provided by PharmApp Solutions GmbH. An overview article provides a general description of Embryotox procedures [6].

For this study, all prospective requests for advice on statin exposure received by Embryotox during the study period from 1 January 2000 to 30 June 2021 were considered. ‘Prospective’ means that neither the pregnancy outcome nor pathological prenatal diagnostics were known at the initial Embryotox consultation. Only pregnancies with statin exposure between gestational week (GW) 2 + 0 days and GW 12 + 6 days were eligible for inclusion. Exposure may have ended in the first trimester or continued beyond. Further criteria of study inclusion are shown in Figure S1. The statin cohort was compared to a randomly selected cohort of prospectively ascertained pregnancies with a completed follow‐up that were not exposed to statins from the Embryotox database. The comparison cohort was matched by year of request at a 1:3 ratio. For both cohorts, cases involving diagnoses of malignancies or maternal co‐medications that are known teratogens or fetotoxicants (with exposure in the second or third trimester) were excluded. Classification of congenital anomalies in all live‐born infants and foetuses was performed according to the EUROCAT‐guideline [7] by two experts blinded to exposure status. Only proven genetic diagnoses were classified as ‘genetic disorder’.

Informed consent was obtained from all individuals included in the study.

### 2.2 Statistical Analysis

The risk ratio (RR) of major birth defects was estimated using log‐binomial regression. Crude rates of major birth defects or genetic disorders were calculated as the number of all infants and foetuses with birth defects divided by the total number of all live‐born infants plus affected foetuses.

Crude rates of pregnancy outcomes (SAB; elective termination of pregnancy—ETOP; stillbirth; live birth) may be biased due to delayed study entry and competing risks [8]. Therefore, cumulative incidences were calculated using event history analysis for cause‐specific sub‐distributions of competing risks while accounting for left truncation due to varying time of gestation at enrolment [8]. The effect of statin exposure on pregnancy outcome was estimated using Cox proportional hazards regression models.

Birth weight and head circumference were evaluated using the German percentile values [9, 10].

To reduce possible confounding, all regression models were adjusted for relevant baseline covariates (see Table S1). In addition, adjustment was made for maternal disease status considering pregestational diabetes, maternal hypertension and renal disorders.

Missing values in the covariate parameters were addressed using multiple imputation. Twenty imputed data sets were generated per outcome. Results were combined using Rubin's rule [11]. All analyses were performed using R version 4.1.2 (R Development Core Team).

## 3 Results

A total of 188 pregnancies met the inclusion criteria for the statin cohort (Figure S1).

### 3.1 Maternal Characteristics

Maternal characteristics of the statin cohort (*n* = 188) and the comparison cohort (*n* = 564) are shown in Table S1. Women in the statin cohort tended to be older (35 vs. 32), to have a lower educational level (academic degree 29.7% vs. 48.6%) and higher nicotine consumption (> 5 cigarettes/day 25.4% vs. 11.1%). The median BMI was higher in the statin cohort (26.6 vs. 22.5), and comorbidities were more common. Additionally, the attitude towards pregnancy was more negative in the statin cohort: 18.5% indifferent and 5.1% not wanted, compared with 6.0% and 1.6%, respectively.

### 3.2 Neonatal Characteristics

The median gestational age at birth was rather similar in both cohorts (GW 38.71 vs. 39.14), but prematurity was more common in the statin cohort (18.5% vs. 11.2%). Newborns in the statin cohort tended to have lower median birth weights (3.110 g vs. 3.325 g) and smaller head circumferences (34 cm vs. 35 cm), see Table S2 for details.

### 3.3 Major Birth Defects

In the statin cohort, 10 out of 135 cases (7.4%) of major birth defects were observed, all of which occurred in live‐born infants, whereas 12 out of 501 (2.4%; RR 3.09, 95% CI 1.37–7.00) were reported in the comparison cohort. After adjustment for relevant baseline covariates (Adjustment 1), the effect estimate was lower (RRadj 2.68, 95% CI 1.14–6.31). After additional adjustment for maternal disease factors (Adjustment 2), the difference further weakened (RRadj 2.16; 95% CI 0.88–5.31); see Table 1. The reported major birth defects were heterogeneous and affected different organ systems (Table S3).

**TABLE 1:** Rate of birth defects.

	Statins (*n* = 188)	Comparison (*n* = 564)	RR (95% CI)	RR adjusted 1 (95% CI)	RR adjusted 2 (95% CI)
Liveborn infants, *n*	135	501			
Major birth defects, *n* (%)	10/135 (7.4)^a^	12/501 (2.4)	3.09 (1.37–7.00)	2.68 (1.14–6.31)	2.16 (0.88–5.31)
Genetic disorders, *n* (%)	3/137 (2.2)^b^	2/501 (0.4)	5.49 (0.93–32.50)	4.16 (0.69–25.09)	n/a

*Note:* RR adjusted 1: adjusted by using propensity scores incorporating the following baseline covariates: maternal age, body mass index (BMI), educational level, smoking habits, alcohol consumption, attitude towards pregnancy, number of previous deliveries, number of previous SAB and number of previous children with birth defects. RR adjusted 2: additionally adjusted for maternal disease status considering pregestational diabetes, maternal hypertension and renal disorders.

Abbreviations: CI, confidence interval; *n*, number of cases [denominators differ because of the different pregnancy outcomes defined as live‐born infants, spontaneous abortion (SAB) or elective termination of pregnancy (ETOP)]; n/a, not applicable; RR, risk ratio.

^a^ All major birth defects were reported in live‐born infants.

^b^ Of these, two foetuses were affected by chromosomal aberrations.

### 3.4 Pregnancy Outcome

In the statin cohort, 133 out of 188 pregnancies resulted in live births, with 135 live‐born infants (including two sets of twins). Twenty‐seven pregnancies ended in SAB, one in stillbirth, and 27 were electively terminated (ETOP); see Table S4. The cumulative incidence of SAB was 26.8% in the statin‐exposed versus 15.0% in the comparison cohort (HR 1.86, 95% CI 1.13–3.04; HRadj 1.66, 95% CI 0.99–2.78; see Figure 1).

**FIGURE 1:**
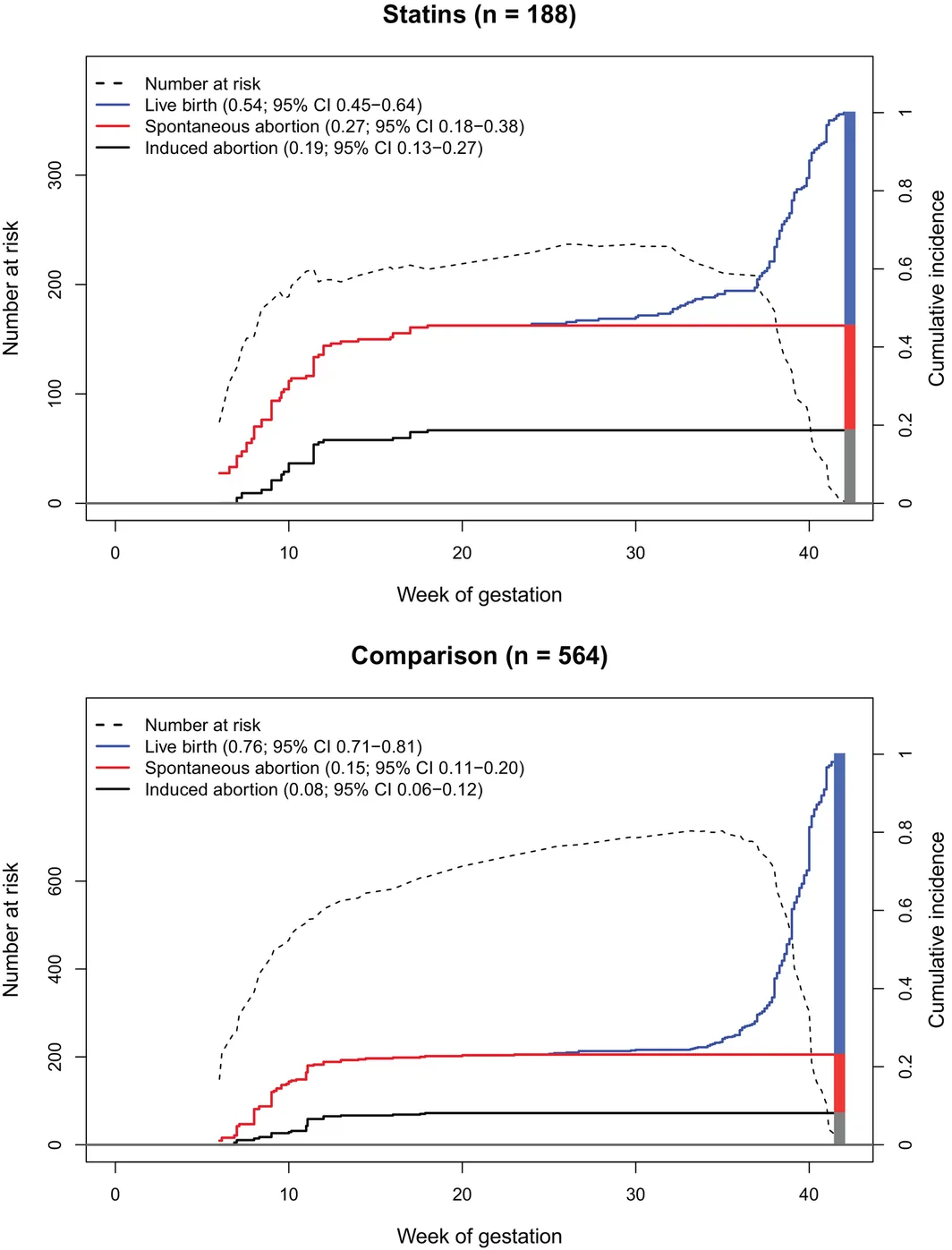
Cumulative incidences of pregnancy outcomes. Cumulative incidences (*y*‐axis on the right) were calculated separately for the statin cohort and the comparison cohort. The dotted line shows the number at risk (*y*‐axis) over time (*x*‐axis) in gestational weeks; *n*, number of cases included.

## 4 Discussion

A strong association between statin exposure and major birth defects is not supported by the present findings, once the adjustments for relevant baseline covariates and maternal disease factors have been applied. As shown in Table S1, the statin‐treated cohort differs from the comparison group in ways other than medication. The statin cohort exhibits specific maternal characteristics and comorbidities that are known to be associated with increased risks of pregnancy and birth defects. These include higher age, obesity, diabetes, hypertension and renal disease. Although the comparison cohort more closely represents the average population, a selection bias may be present [12]. However, the study and comparison cohorts were taken from the same Embryotox data pool and matched appropriately. The rate of major birth defects in the comparison cohort is within the range of previously published prevalence rates [13, 14]. Given the small number of major birth defects in the limited sample size, random differences cannot be ruled out.

Published meta‐analyses consistently found no significant association between statin exposure during pregnancy and overall rate of major congenital anomalies [1, 2, 3]. However, the most critical factor is confounding by underlying maternal disease. Bateman et al. demonstrated this impact directly in their cohort study: unadjusted analysis using US Medicaid data showed an elevated risk (RR 1.79, 95% CI 1.43–2.23), but after controlling for confounders, particularly pre‐existing diabetes, the risk attenuated to non‐significance (RR 1.07, 95% CI 0.85–1.37) [15].

Findings for cardiac defects are more ambiguous (e.g., [2, 16]). Even in studies showing statistically significant associations (e.g., for ventricular septal defects, see [16]), the absolute increase is modest from a clinical viewpoint. A recent Norwegian national study using linked data from four national health registries examined over 800 000 pregnancies and found no significant association (ORadj 1.30, 95% CI 0.81–2.09) for any congenital malformations. Their updated meta‐analysis suggested no increased cardiac malformation risk (OR 1.24, 95% CI 0.94–1.64) [1]. The cohort study described here involved a detailed evaluation of major birth defects, which affected various organ systems (see Table S3). Congenital heart defects were observed in 4/10 infants, thereof three cardiac septal defects, known to be the most frequently observed major birth defects in humans [14].

Two meta‐analyses showed elevated risks for SAB, with an OR of 1.5 (95% CI 1.1–2.0) and 1.36 (95% CI 1.06–1.75) [2, 3]. These took into account the same three or four cohort studies, respectively. One of the included observational multicentre studies, however, found no significant difference in the SAB rate after adjusting for maternal age and gestational age (HR 1.36, 95% CI 0.63–2.93) [17]. In our study, the cumulative incidence of SAB was 26.8% in the statin‐exposed cohort (HRadj 1.66, 95% CI 0.99–2.78). Furthermore, it is important to note that ETOP occurred more frequently in the statin cohort as a competing event (18.7%, HRadj 1.55, 95% CI 0.83–2.89). In most cases, the decision to undergo ETOP was made for personal reasons (Table S4). Overall, as shown in Table S1, the attitude towards pregnancy was more negative in the statin cohort.

Premature birth was more common in the statin‐exposed cohort evaluated here (18.5% vs. 11.2%), and the infants tended to have a lower birth weight and smaller head circumference (Table S2). Other studies reported similar trends [17, 18, 19]. The associations with preterm birth and low birth weight likely reflect the underlying maternal disease issues rather than direct statin effects, though distinguishing these mechanisms is difficult.

## 5 Conclusions

The most plausible interpretation of the study results is that statins are unlikely to be the primary factor responsible for adverse pregnancy outcomes. In fact, women who require statins have certain characteristics that may increase the risk of birth defects and affect neonatal outcome. This is also supported by our study results after adjustment. Existing evidence suggests that unintended exposure to statins in early pregnancy is unlikely to cause harm to the developing embryo. A critical risk–benefit assessment and an individual decision are essential when considering whether to continue statin therapy in high‐risk patients. In both situations, a second‐trimester ultrasound scan should be offered.

## Funding

This work was supported by the German Federal Institute for Drugs and Medical Devices (BfArM) (FKZ V‐2020.10/68605/2022‐2025). The funder had no role in study design, data collection and analysis, decision to publish or preparation of the manuscript.

## Ethics Statement

Ethical approval was granted by the Ethics Committee of Charité—Universitätsmedizin Berlin, Germany (EA2/011/22). This study was registered in the German Clinical Trials Register.

## Conflicts of Interest

The authors declare no conflicts of interest.

## Supporting information


**Figure S1:** Flow chart.
**Table S1:** Maternal characteristics of study cohorts.
**Table S2:** Neonatal characteristics.
**Table S3:** Major birth defects reported in the statin exposed cohort.
**Table S4:** Pregnancy outcomes.

## Data Availability

The data that support the findings of this study are available on request from the corresponding author. The data are not publicly available due to privacy or ethical restrictions.
