# Supplementary material for: Pregnancy Outcome After First‐Trimester Exposure to Statins: Study Results Based on the Embryotox Cohort
Source: Pharmacoepidemiol Drug Saf. 2026 Sep 28;35(10):e70481. doi: 10.1002/pds.70481 (PMC13618450; doi:10.1002/pds.70481)
Supplement: Supplementary file 1 [file PDS-35-e70481-s001.pdf]

Supporting Information

Pregnancy outcome after first trimester exposure to statins – study results based on the Embryotox cohort

Figure S1. Flow chart

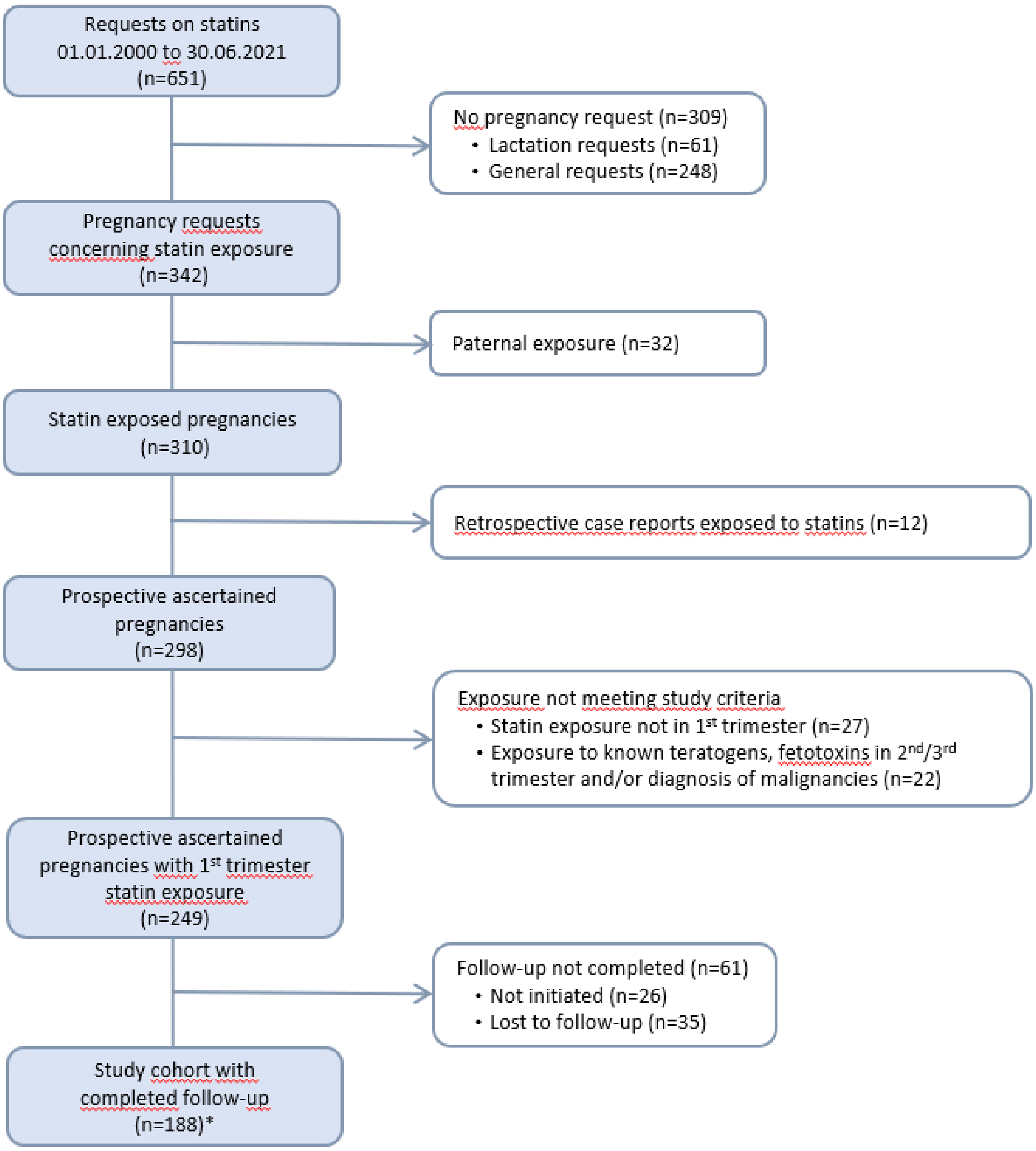

Legend. Procedure for selecting the final study cohort from all consultation requests * Including study cases exposed to simvastatine (n=108), atorvastatine (n=49), pravastatine (n=13), fluvastatine (n=11), rosuvastatine (n=4), cerivastatine (n=3). Three cases (atorvastatine, pravastatine, fluvastatine) switched to simvastatine after recognition of pregnancy.

Table S1. Maternal characteristics of study cohorts

	Statins	Comparison
n	188	564
**Age*, n**	188	562
Age (years)	35 (30-38) (16-45)	32 (28-35) (17-60)
**BMI*, n**	143	455
BMI	26.6 (22.9-32.6) (17.3-59.8)	22.5 (20.6-26) (16.3-50.1)
**Educational level*, n**	91	333
No leaving exam (<9 years), n %	3 (3.3)	2 (0.6)
9 years exam, n %	15 (16.5)	23 (6.9)
10/11 years exam, n %	29 (31.9)	81 (24.3)
Secondary school exam, n %	17 (18.7)	65 (19.5)
Academic degree, n %	27 (29.7)	162 (48.6)
**Smoking*, n**	181	557
No	125 (69.1)	472 (84.7)
<= 5 cig/day	10 (5.5)	23 (4.1)
> 5 cig/day	46 (25.4)	62 (11.1)
**Alcohol*, n**	180	557
No, n %	167 (92.8)	523 (93.9)
≤ 1 drink/day, n %	10 (5.6)	21 (3.8)
> 1 drink/day, n %	3 (1.7)	13 (2.3)
**Pregnancy wanted*, n**	157	487
Yes, n %	120 (76.4)	450 (92.4)
Indifferent, n %	29 (18.5)	29 (6)
No, n %	8 (5.1)	8 (1.6)
**Previous pregnancies, n**	185	557
0, n %	77 (41.6)	244 (43.8)
1, n %	39 (21.1)	163 (29.3)
2, n %	40 (21.6)	90 (16.2)
3 or more, n %	29 (15.7)	60 (10.8)
**Previous deliveries*, n**	185	558
0, n %	87 (47)	292 (52.3)
1, n %	47 (25.4)	184 (33)
2, n %	38 (20.5)	59 (10.6)
3 or more, n %	13 (7)	23 (4.1)
**Previous SAB*, n**	184	556
0, n %	156 (84.8)	457 (82.2)
1, n %	16 (8.7)	69 (12.4)
2 or more, n %	12 (6.5)	30 (5.4)

**Previous children with birth defect*, n**	183	556
0, n %	178 (97.3)	546 (98.2)
1, n %	5 (2.7)	8 (1.4)
2 or more, n %	0 (0)	2 (0.4)
**Maternal comorbidities**, n**	188	564
Pregestational diabetes, n (%)	14 (7.4)	4 (0.7)
Hypertension, n (%)	55 (29.3)	37 (6.6)
Renal disorder, n (%)	14 (7.4)	2 (0.4)
**First contact for counselling to Embryotox, n**	188	564
GW at first contact	8 (6-10.9) (2.9-40)	8.6 (6.3-14.2) (2.7-40.9)

Legend. Median, interquartile range and min/max are presented for maternal age, BMI and GW at first contact to the Embryotox Centre. BMI, body mass index in kg/m^2; GW, gestational week; SAB, spontaneous abortion; n, number of cases.

* Baseline covariates used for adjustment 1. ** Additional covariates used for adjustment 2.

Table S2. Neonatal characteristics

	**Statins**	**Comparison**
N	135	501
**Gestational week, n**	135	501
GW at birth	38.71 (37.4-40) (24-42)	39.14 (38-40) (25-42)
**Preterm Birth, n**	135	501
Preterm	25 (18.5)	56 (11.2)
**Child's sex, n**	135	501
Female	72 (53.3)	230 (45.9)
Male	63 (46.7)	271 (54.1)
**Weight, n**	134	501
Child weight	3110 (2505-3585) (442-5160)	3325 (3020-3650) (450-5220)
**Length, n**	133	495
Child length	50 (47-52) (27-58)	51 (50-53) (29-60)
**Head circumference, n**	122	454
Head circumference	34 (32-35.875) (20.3-38)	35 (34-36) (19.5-39)

Legend. Neonatal characteristics (including twins). For gestational week at birth, weight and length of the baby, median, interquartile range and min/max are presented. GW, gestational week; n, number of infants; preterm, <37+0 gestational weeks at birth.

Table S3. Major birth defects reported in the statin exposed cohort

Case	Exposure to statins (GW)	Substance	GW at birth*, weight, sex	Major birth defect (additional relevant information)
1	0 – 16	Cerivastatine	GW 39, 50-75% p., female	Severe foot deformity (surgery planned)
2	0 – 6	Simvastatine	GW 39, 75-90% p., male	Renal hypoplasia (dysmorphic features, cytogenetics: balanced autosomal translocation)
3	0 – 8	Pravastatine	GW 38, 10-25% p., male	Complex heart defect
4	0 – 9	Fluvastatine	GW 33, 25-50% p., female	Atrial septal defect, mild pulmonary artery stenosis
5	0 – 6	Fluvastatine	GW 28, 10-25% p., female	Atrial septal defect
6	0 – 8	Fluvastatine	GW 36, 3-10% p., female	Multiple limb anomalies, cleft palate (dysmorphic features)
7	0 – 7	Simvastatine	GW 41, 50-75% p., female	Cleft lip and palate
8	0 – 8	Simvastatine	GW 40, <3% p., female	Microcephaly
9	0 – 7	Atorvastatine	GW 41, 75-90% p., male	Atrial septal defect (abnormal hearing test)
10	0 – 6	Pravastatine	GW 41, 3-10% p., male	Microcephaly

Legend. *All were live-born infants. GW, gestational week; p., percentiles

Table S4. Pregnancy outcomes

	Statins n (%)	Comparison n (%)	HR (95% CI)	HR adjusted 1 (95% CI)	HR adjusted 2 (95% CI)
**Pregnancies, n**	188 [a]	564 [b]			
**Outcomes, n**	190	574			
**SAB, n (%)**	27 (14.4)	41 (7.3)	1.86 (1.13-3.04)	1.67 (1.01-2.77)	1.66 (0.99-2.78)
**ETOP, n (%)**	27 (14.4)	28 (5.0)	2.87 (1.69-4.88)	1.61 (0.88-2.97)	1.55 (0.83-2.89)
- personal decision	17 (9.0)	22 (3.9)			
- embryopathology	2 (1.1)	-			
- medical reasons	7 (3.7)	5 (0.9)			
- unknown	1 (0.5)	1 (0.2)			
**Stillbirth, n (%)**	1 (0.5)	4 (0.7)	0.96 (0.11-8.58)	1.00 (0.11-9.03)	0.51 (0.05-5.30)
**Livebirth, n (%)**	133 (70.7)	493 (87.4)	1.13 (0.93-1.36)	1.09 (0.88-1.33)	1.02 (0.83-1.25)
**Liveborn infants, n**	135	501			

Legend. CI, confidence interval; ETOP, elective termination of pregnancy; HR, hazard ratio; n, number of cases; SAB, spontaneous abortion. [a]including two twin pregnancies with live-born infants. [b]including eight twin pregnancies with live-born infants, and two twin pregnancies resulting in one SAB and one live-born infant.

HR adjusted 1: adjusted by incorporating the following baseline covariates: maternal age, body mass index (BMI), educational level, smoking habits, alcohol consumption, attitude towards pregnancy, number of previous deliveries, and number of previous SAB;

HR adjusted 2: additionally adjusted for maternal disease status considering pregestational diabetes, maternal hypertension and renal disorders.
